# Integrated Genetic and Epigenetic Analysis Identifies Haplotype-Specific Methylation in the *FTO* Type 2 Diabetes and Obesity Susceptibility Locus

**DOI:** 10.1371/journal.pone.0014040

**Published:** 2010-11-18

**Authors:** Christopher G. Bell, Sarah Finer, Cecilia M. Lindgren, Gareth A. Wilson, Vardhman K. Rakyan, Andrew E. Teschendorff, Pelin Akan, Elia Stupka, Thomas A. Down, Inga Prokopenko, Ian M. Morison, Jonathan Mill, Ruth Pidsley, International Type 2 Diabetes 1q Consortium, Panos Deloukas, Timothy M. Frayling, Andrew T. Hattersley, Mark I. McCarthy, Stephan Beck, Graham A. Hitman

**Affiliations:** 1 Medical Genomics, UCL Cancer Institute, University College London, London, United Kingdom; 2 Blizard Institute of Cell and Molecular Science, Barts and The London School of Medicine and Dentistry, Queen Mary University of London, London, United Kingdom; 3 Wellcome Trust Centre for Human Genetics, University of Oxford, Oxford, United Kingdom; 4 Wellcome Trust Sanger Institute, Hinxton, United Kingdom; 5 Comparative Genomics, UCL Cancer Institute, University College London, London, United Kingdom; 6 Gurdon Institute, University of Cambridge, Cambridge, United Kingdom; 7 Department of Genetics, University of Cambridge, Cambridge, United Kingdom; 8 Pathology Department, Dunedin School of Medicine, University of Otago, Dunedin, New Zealand; 9 Institute of Psychiatry, King's College London, London, United Kingdom; 10 Institute of Biomedical and Clinical Science, Peninsula Medical School, University of Exeter, Exeter, United Kingdom; Institute of Preventive Medicine, Denmark

## Abstract

Recent multi-dimensional approaches to the study of complex disease have revealed powerful insights into how genetic and epigenetic factors may underlie their aetiopathogenesis. We examined genotype-epigenotype interactions in the context of Type 2 Diabetes (T2D), focussing on known regions of genomic susceptibility. We assayed DNA methylation in 60 females, stratified according to disease susceptibility haplotype using previously identified association loci. CpG methylation was assessed using methylated DNA immunoprecipitation on a targeted array (MeDIP-chip) and absolute methylation values were estimated using a Bayesian algorithm (BATMAN). Absolute methylation levels were quantified across LD blocks, and we identified increased DNA methylation on the *FTO* obesity susceptibility haplotype, tagged by the rs8050136 risk allele A (*p* = 9.40×10^−4^, permutation *p* = 1.0×10^−3^). Further analysis across the 46 kb LD block using sliding windows localised the most significant difference to be within a 7.7 kb region (*p* = 1.13×10^−7^). Sequence level analysis, followed by pyrosequencing validation, revealed that the methylation difference was driven by the co-ordinated phase of CpG-creating SNPs across the risk haplotype. This 7.7 kb region of haplotype-specific methylation (HSM), encapsulates a Highly Conserved Non-Coding Element (HCNE) that has previously been validated as a long-range enhancer, supported by the histone H3K4me1 enhancer signature. This study demonstrates that integration of Genome-Wide Association (GWA) SNP and epigenomic DNA methylation data can identify potential novel genotype-epigenotype interactions within disease-associated loci, thus providing a novel route to aid unravelling common complex diseases.

## Introduction

Type 2 diabetes (T2D) and obesity are complex diseases with polygenic susceptibility. Recent genome wide association (GWA) studies have provided significant insight towards gene discovery and, to date, around 30 common variant loci have been associated with T2D [1], [2]. Despite these important advances, known genetic variants explain <10% of the heritable component of disease, and little is known about their contribution to aetiology. Ongoing studies are attempting to explain this ‘missing heritability’ in complex diseases via the detection of rare and structural variants, interactions between discovery SNPs and causal variants, and identification of associated stable epigenetic modifications [3].

Environmental determinants of these complex diseases are well-characterised by a wealth of epidemiological literature [4] but much less is understood about their mechanistic role or interaction with genetic susceptibility. Epigenetic mechanisms regulate gene expression, may be induced via environmental agents and can be inherited through somatic or germline pathways [5]. Genome-wide epigenetic reprogramming occurs during gametogenesis, implicating early fetal development as a period susceptible to this environmental influence. Strong evidence in humans and rodents implicates *in utero* fetal nutritional deficiency with adult-onset chronic diseases including T2D [6], [7]. Furthermore, differentially methylated regions (DMRs) of genes of relevance to T2D have been identified in individuals prenatally exposed to famine [8]. Epigenetic variability, set at an extremely early development stage, permeates through all three subsequently developing germ layers [9], thereby facilitating possible identification in mesoderm-derived blood.

An epigenomic approach may provide insight into the genetic, epigenetic and environmental factors underlying the aetiology of complex diseases. The potential for interaction between these factors is increasingly understood, however the relationship between specific epigenetic modifications and genomic features, whether independent or dependent, is poorly explained in the context of disease and phenotype. In contrast, the role of aberrant epigenetic marks is well established at single gene loci with rare imprinted disorders [10] and as well in cancer genomes with global hypomethylation and locus hypermethylation [11]. Recent investigations in other complex diseases, such as Systemic Lupus Erythematosus with discordant monozygotic twins [12] and Type 1 diabetes with imprinted common susceptibility [13], have been successful. A range of new technology platforms and bioinformatic tools now exist with which to test hypotheses around these putative interactions. The developmental and environmental origins of T2D suggest a potential role of programmed and/or stochastic epigenetic processes in determining disease risk that could arise during early embryonic development and are identifiable in a range of tissues. We hypothesize that genotype-epigenotype interactions may underlie the aetiopathogenesis of T2D, as a complex disease, and use an integrated platform with which to investigate their occurrence. We applied methylated DNA immunoprecipitation (MeDIP) to fragmented genomic DNA sampled from participants with and without T2D-risk genotypes. Enriched fragments were hybridised against total input DNA on a Nimblegen 385k array, encompassing known regions of genetic susceptibility to T2D (regions identified from previous locus and genome-wide association analyses; monogenic T2D and obesity genes and related disorders (as of 2008) (see Supplementary Table S1 for full list). With each array tiling >21 Mb, a total of >1.2 Gb of sequence was subsequently statistically assessed for DNA methylation status. By combining genotype with methylation status, an investigation for allele- [14] or haplotype-specific effects and hepitypes [15], [16], [17] could be made to understand the potential sources of methylation variability at these genomic locations.

## Results

We determined DNA methylation differences on DNA derived from peripheral whole blood from 60 Caucasians, comprising 30 with Type 2 diabetes (UK Warren 2 T2D case sample) and 30 without T2D (Exeter Family Study of Childhood Health). An all-female set was used to remove any confounding effect of sex.

### DNA Methylation Analysis within T2D Association SNP LD Blocks

MeDIP-chip was performed on individual samples to quantify DNA methylation. The relationship between genetic haplotypes and DNA methylation at the genomic regions covered by the microarray was estimated by summating the methylation scores, estimated by the BATMAN algorithm, across each T2D association SNP LD block (as defined by Gabriel *et al*. [18]), and calculating an average ‘methylation load’ for all 60 individuals. Individuals then were grouped into three genotypic sets by their respective genotype for each T2D-association SNP (or tagging SNP in perfect LD with association SNP). Kruskal-Wallis and Linear Regression analyses were performed to determine the relationship between genotype and average methylation score. Uncorrected *p*-values for the Linear Regression analysis are shown in Table 1.

**Table 1:** Average Methylation in Association SNP LD blocks by Genotype.

					Average Methylation			
Chr	LD Block		Genotyped SNP	Gene/Locus	11	12	22	*p*-value
1	120236149	120398430	rs2934381	*NOTCH2*	0.496	0.495	0.502	0.187
2	43529937	43617946	rs7578597	*THADA*	0.492	0.512	0.504	0.564
3	12298413	12372392	rs1801282	*PPARG*	0.512	0.509	0.511	0.640
3	64673853	64705161	rs4607103	*ADAMTS9*	0.477	0.472	0.481	0.760
3	186971576	187031377	rs4402960	*IGF2BP2*	0.502	0.493	0.502	0.016
4	6317902	6363877	rs10010131	*WFS1*	0.581	0.604	0.590	0.982
7	28147081	28175361	rs864745	*JAZF1*	0.501	0.492	0.494	0.317
8	118252732	118254914	rs13266634	*SLC30A8*	0.333	0.350	0.303	0.865
9	22122209	22126489	rs10811661	*CDKN2A/CDKN2B*	0.543	0.512	0.512	0.389
10	12367941	12368040	rs12779790	*CDC123/CAMK1D*	0.611	0.590	0.671	0.913
10	94426831	94467199	rs1111875	*HHEX*/*IDE*	0.480	0.483	0.483	0.369
11	17350649	17365206	rs5219	*KCNJ11*	0.501	0.502	0.498	0.540
12	69942990	69949369	rs7961581	*TSPAN8*	0.457	0.461	0.470	0.289
16	52357008	52402988	rs8050136	*FTO*	0.497	0.510	0.531	9.397′10^−4^
17	33170413	33182480	rs757210	*HNF1B*	0.423	0.430	0.427	0.382

*P*-values are calculated by Linear Regression and are shown uncorrected (11 – homozygote common, 12 – heterozygote, 22 – homozygote rare allele).

a) r^2^ = 1 with rs10923931,

b) Not in LD block – single BATMAN window of 100 bp utilised,

c) LD block of associated SNP rs5215 used as rs5219 not typed in HapMap, r^2^ = 0.995 with rs5219. Methylation is given as average BATMAN scores across regions (0 =  unmethylated, 1 =  fully methylated).

### Sliding Windows and Permutation Methylation Analysis of the *FTO* LD Block

**Figure 1:**
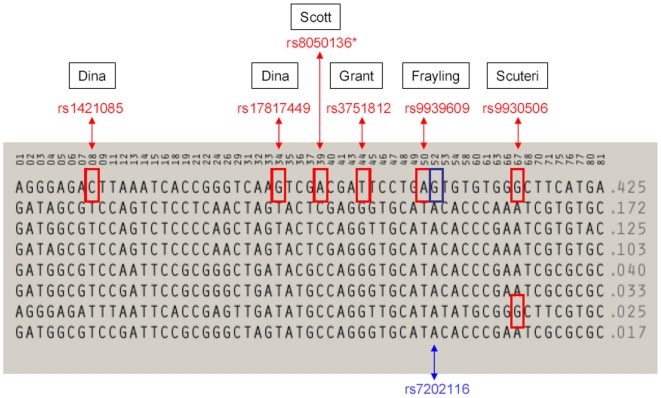
Haplotypes for *FTO* Susceptibility LD Block from HapMap CEU. BMI Association-SNPs are in red boxes for the respective studies. SNPs 08 (rs1421085) & 34 (rs17817449) – Dina *et al*. [33], SNP 39 (rs8050136) – Scott *et al*. [34], SNP 44 (rs3751812) – Grant *et al*. [65], SNP 50 (rs9939609) – Frayling *et al*. [22], SNP 67 (rs9930506) – Scuteri *et al*. [35]. *The SNP rs8050136 genotypes were used as the haplotype tagging SNP for this study's subjects. SNP 52 – (rs7202116 blue box) is CpG-creating or abrogating dependent upon which allele is present and resides within the 900 bp window peak.

In our integrated epigenomic-genomic analysis, the large 46 kb LD block of the *FTO* gene (Supplementary Figure S1) was the only locus to reach nominal statistical significance by Kruskal-Wallis analysis (*p* = 0.014). We performed a permutation analysis, in which the genotype assignment and observed methylation scores were shuffled 10,000 times, achieving a significant empirical *p*-value of 0.012. Subsequent analysis by Linear Regression was highly significant with *p* = 9.40×10^−4^, and empirical *p*-value calculation by 10,000 permutations of 1.0×10^−3^. Age was included as a factor in this later analysis, as methylation changes due to age are significant in certain loci [19], [20], [21], but was excluded as a significant confounder (*p* = 0.676). We also performed linear regression analysis for the other tested loci and did not find any association between age and methylation (data not shown). *FTO* SNP rs8050136 risk allele A homozygotes were shown to have a higher average level of methylation of 0.531, with heterozygotes at an intermediate level at 0.510 and G homozygotes with 0.497 (Table 2). Of note, although the initial association for *FTO* with BMI was identified by SNP rs9939609 [22], numerous subsequent studies have used different SNPs, all of which capture the same common haplotype (HapMap CEU frequency  = 0.425 (Figure 1)). This LD Block encompasses just under half of the first intron, exon 2 and the beginning of the second intron of the major *FTO* isoform (Supplementary Figure S1).

**Table 2:** Average Methylation by rs8050136 Genotype.

	*FTO* LD Block(46 kb)	Broad Peak(7.7 kb)	Narrow Peak(900 bp)
Region	52,357,008–52,402,988	52,371,700–52,379,399	52,378,500–52,379,399
AA	0.531	0.529	0.603
AC	0.510	0.503	0.564
CC	0.497	0.478	0.507
*p*-value	9.397×10^−4^	1.133×10^−7^	1.94×10^−5^

**Figure 2:**
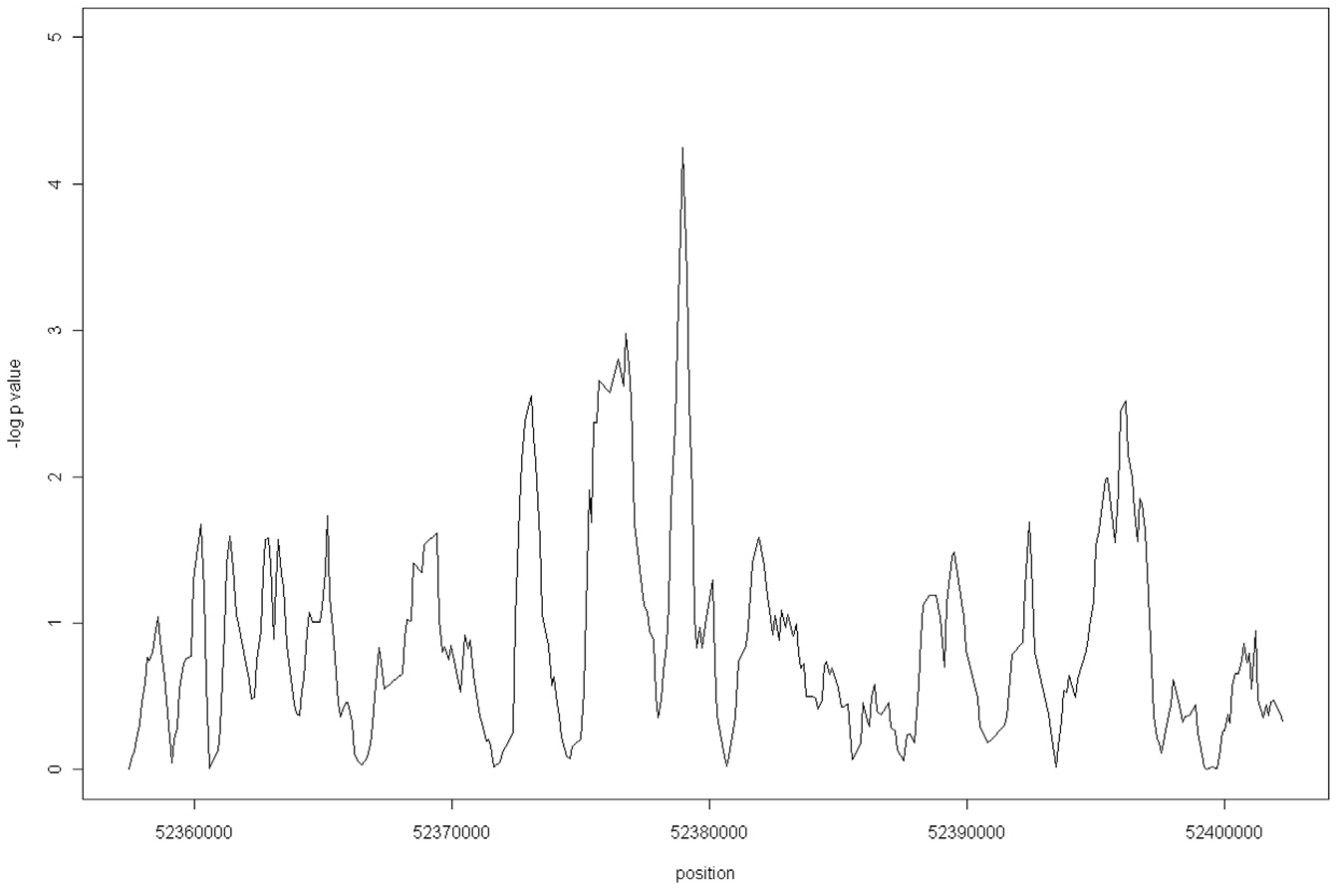
Linear Regression for Methylation with respect to Genotype for 9 Window. Linear regression *p*-values (-log _10_) for a sliding window of 9 BATMAN windows across the *FTO* LD susceptibility block (Chr16:52,357,008–52,402,988)

**Figure 3:**
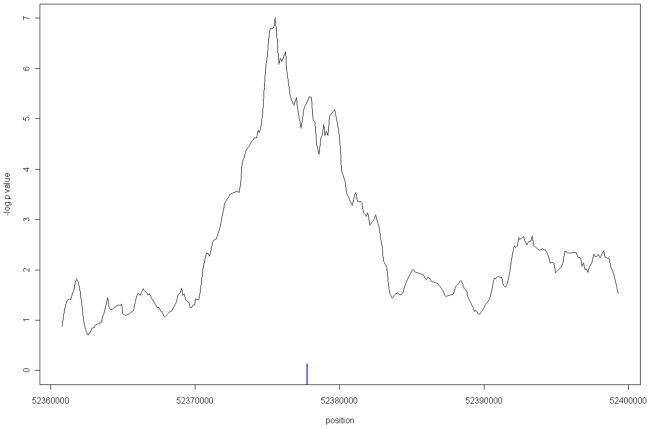
Linear Regression for Methylation with respect to Genotype for 60 Window. Linear regression p-values (-log _10_) for a sliding window of 60 BATMAN windows across the *FTO* LD susceptibility block (Chr16:52,357,008–52,402,988). Blue bar indicates location of HCNE enhancer element 6 from Ragvin *et al*. [30].

Results shown for the entire LD block, the Broad 7.7 kb 60-window peak (Figure 3) and the Narrow 900 bp 9-window peak (Figure 2). Methylation is given as average BATMAN scores across regions (0 =  unmethylated, 1 =  fully methylated).

To investigate whether this methylation difference was being driven by a distinct region within the 46 kb LD block, and if so, to isolate its location, a sliding windows analysis utilising BATMAN methylation scores (estimated in 100 bp windows) was performed. The sliding windows analysis uses a computational approach to move different window sizes across each LD block, starting with a window size of 1 (100 bp), increasing iteratively by one on each pass, up to the maximum window number of 334 (Supplementary Videos S1 and S2). A central peak of methylation difference was most prominent in the 9 window (900 bp wide, at windows 161–169, chr16:52,378,500-52,379,399) analysis with Kruskal-Wallis *p* = 5.630×10^−5^, empirical *p* = <1×10^−5^ with 10,000 permutations and Linear Regression analysis *p* = 1.94×10^−5^ (Figure 2). Methylation averages were again highest for the A homozygotes (AA 0.603, AC 0.564, CC 0.507). In addition, plotting the slope of linear regression analysis for the 9 window across the LD block indicated that the *p*-value peaks all co-locate with the same negative regression slope peaks. From these findings, we infer that all of these regions could be contributing, with varying strength, to the increased methylation signal (Supplementary Figure S2).

**Figure 4:**
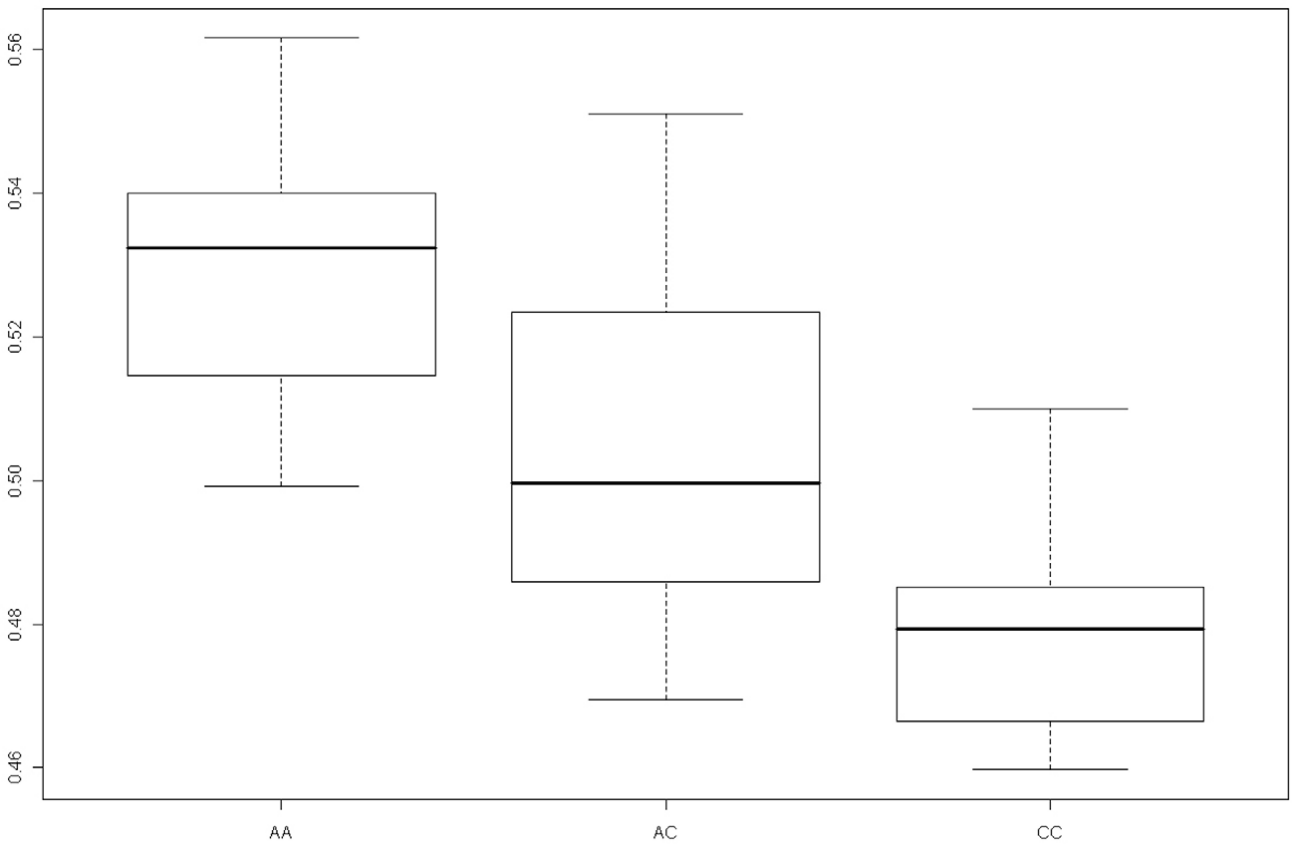
Box plot of Methylation Scores with respect to Genotype in 7.7 kb Window. Methylation values for Broad Peak within 7.7 kb window for AA susceptibility homozygote, AC heterozygote and CC homozygote of rs8050136. Linear regression *p* = 1.33×10^−7^.

The strongest association between methylation and genotype was identified with a larger window size of 60 (7.7 kb, after exclusion of repeat regions, - windows 110–169, chr16:52,371,700–52,379,399) with Kruskal-Wallis *p* = 8.69×10^−6^, empirical *p* = <1×10^−5^ with 10,000 permutations, Linear Regression analysis *p* = 1.33×10^−7^ (Figure 3) and age *p* = 0.444. This 7.7 kb window becomes most significant just at the point when it encapsulates the 900 bp window at its downstream edge. The same trend in the average methylation scores was again identified (AA 0.529, AC 0.503, CC 0.478, Figure 4). The sliding windows analysis was also performed across the other T2D association LD blocks, but none showed any significant increasing *p*-values with window sizes greater than 10 (1 kb) (data not shown).

The findings at the *FTO* LD block, using an integrated epigenomic-genomic analysis, give a strong estimate of the relationship between haplotype and DNA methylation level. Since this approach is calculated by statistically-derived methylation estimates in 100 bp windows, it is unable to define the actual methylation state neither of individual CpG sites nor their relationship to SNPs. We therefore proceeded to investigate the genetic and epigenetic architecture of the 900 bp peak window to explore the exact methylation differences driving this signal.

### Investigation of the Genetic Architecture Underlying the 900 bp Window Peak

The central 9 window peak of 900 bp contains only seven CpG sites in the reference sequence, although of the eight common SNPs within the same region, three create or abrogate additional CpG sites (being YpG or CpR SNPs, IUPAC: Y = C or T, R = G or A). These SNPs (rs7206629, rs7202116 and rs7202296) were all included in the AFD-EUR European panel (dbSNP). In this dataset they all possess identical minor allele frequencies (MAFs) implying high likelihood of residing on the same haplotype (Table 3). Only rs7202116 is included in the HapMap CEU data set, and the methylated state allele, G of a CpG, is present on the susceptibility haplotype (Figure 1), and is in absolute and near-perfect LD with the susceptibility SNPs identified (Table 4). Therefore those with the obesity-susceptibility haplotype would possess ten CpG sites capable of methylation, compared with seven, within this peak region.

**Table 3:** Allele Frequencies for CpG-creating SNPs in 900 bp window peak.

SNP		Major	Minor	EUR		CHN		AFR		Ancestral
rs7206629	YpG	T	**C**	0.609	0.391	0.833	0.167	0.587	0.413	C
rs7202116	CpR	A	**G**	0.609	0.391	0.833	0.167	0.674	0.326	A
rs7202296	CpR	A	**G**	0.609	0.391	0.833	0.167	0.674	0.326	A

CpG creating allele given in bold. EUR  =  European, CHN  =  Asian, AFR  =  African American from dbSNP. CpG-creating sites (YpG or CpR SNPs, IUPAC: Y = C or T, R = G or A).

**Table 4:** LD relationship for *FTO* Association SNPs and rs7202116.

					r^2^			
	SNP	rs1421085	rs17817449	rs8050136	rs3751812	rs9939609	rs7202116*	rs9930506
	rs1421085		0.927	0.931	0.931	0.931	0.965	0.835
	rs17817449	0.963		1	1	1	0.964	0.833
D'	rs8050136	0.965	1		1	1	0.966	0.841
	rs3751812	0.965	1	1		1	0.966	0.841
	rs9939609	0.965	1	1	1		0.966	0.841
	rs7202116*	1	1	1	1	1		0.871
	rs9930506	0.963	0.962	0.964	0.964	0.964	1	

Results given for D' and r^2^ in CEU HapMap population. *Indicates the methylation critical SNP within 900 bp window peak.

### Validation of Methylation Differences by Pyrosequencing

Methylation differences identified between the susceptibility and non-susceptibility haplotypes were validated and quantified using bisulphite-pyrosequencing on 80 samples, using the initial 60 plus an additional 20 samples from the same studies. Within the 900 bp window, four CpGs were investigated, located at the downstream end of the 7.7 kb peak, and included one of the SNP dependent CpGs (rs7202296). An additional six CpGs that lie directly beneath the broad 7.7 kb peak, including one SNP dependent CpG (rs11075988) were examined. The common SNP distribution within the 7.7 kb region, which includes the 900 bp peak, is detailed in Supplementary Figure S4.

Methylation levels at the cytosine adjacent to rs7202296 (CpR) were, as expected, completely dependent on the presence of its CpG conformation, with average methylation levels of 87% for A homozygotes, 55.7% for AC heterozygotes and 11.5% for C homozygotes, with respect to rs8050136 genotype (Table 5). On examination of the sequence information from the pyrosequencing data, due to non-perfect LD between rs8050136 and rs7202296, one individual homozygous for the A rs8050136 allele was in fact homozygous CpA and another was heterozygote. Excluding these two individuals, the methylation level was 98.6% for those who were truly genetically CpG at rs7202296. The additional cytosines examined within this window did not show differences that could be accounted for by allele-specific methylation (ASM) or the presence of a hepitype, whereby a *cis*-methylation effect on the surrounding non-polymorphic CpG's methylation state would have been observed [15]. Of note is the ∼50% methylation values of the cytosine at 52,379,254, implying that whilst no evidence of ASM, there may be parent-of-origin specific methylation imprinting of this CpG, although *FTO* is not known to be imprinted [23]. The three in-phase CpG-creating SNPs could easily account on their own for the ∼10% difference seen in methylation levels in the 900 bp window peak identified in the initial MeDIP-array. The additional CpGs beneath the peak of Window 60, also showed methylation changes associated with a CpR site at rs11075988. At this SNP, the allele that abrogated the ability to methylate was in LD with the rs8050136 susceptibility genotype A, *i*.*e*. it was on the alternate phase. Decreased methylation of this one SNP is therefore vastly outweighed by the rest of the haplotype. The additional six CpGs showed methylation percentages for AA, AC and CC genotypic groups respectively of 77.6, 72.8, 76.7 (16:52377247), 92.7, 92.0, 90.8 (16:52377254), 9.5, 38.4, 69.0 (16:52377271, rs11075988 dependent), 96.7, 98.5, 98.8 (16:52377279), 82.6, 78.4, 81.1 (16:52377282), and 87.4, 85.9, 84.9 (16:52377308).

**Table 5:** Pyrosequencing Validation Assay of Bisulphite-treated DNA – Cytosine Methylation %.

	CpG Location			
rs8050136Genotype	52379190*	52379221	52379251	52379254
AA	87	95.1	96.7	49.6
AC	55.7	95.5	97	51.6
CC	11.5	95.1	96.6	49.9

Location of methylated Cytosine of CpG from Hs36 Build within 900 bp 9-Window.

*CpG-creating SNP rs7202296 dependent.

### Replication of Findings in Central Nervous Tissue

Finally, we used the same bisulphite-pyrosequencing assays to determine whether methylation differences within the 900 bp window peak were displayed in tissues which show high *FTO* expression and have a role in central energy balance. We studied 14 healthy brain samples (4 hypothalamus and 10 prefrontal cortex) and replicated the same findings at the CpG-creating SNPs described above. We did not see any allele-specific effects on the surrounding CpG sites.

### Evolutionary Analysis

Examination of the three critical CpG-creating SNPs within the narrow peak (rs7206629, rs7202116 and rs7202296) revealed that the latter two have transitioned from CpA to CpG, gaining the ability to methylate from the ancestral state (identical in *Pan Troglodytes*, *Pongo pygmaeus* and *Macaca mulatta*, Table 6). This mutational event is rarer than the reverse transition by deamination of the methylated cytosine [24], and identical frequency of both SNPs are seen in three ethnicity panels (Table 3). Analysis of dbSNP data (release 129) indicated that whilst ∼60% of 193,133 validated SNPs on chromosome 16, with known ancestral state, occur at a CpG site, only ∼12% of them have gained the ability to methylate since their common ancestor and less than 0.5% lie within at least 75 bp of each other, as per rs7202116 and rs7202996. Furthermore, rs7202116 is located within a short 49 bp mammalian evolutionarily constrained element (Chr16:52,379,085–52,379,132) as identified by Genomic Evolutionary Rate Profiling (GERP) [25] of 31 eutherian mammals.

**Table 6:** Primate Comparison for 900 bp CpG-creating SNPs.

	rs7206629	rs7202116	rs7202296
*Homo sapiens*	…TTGGT**Y**GAAGT…	…TAAAC**R**TCTTT…	…AAGCC**R**ATAAA…
*Pan troglodytes*	…TTGGTCGAAGT…	…TAAACATCTTT…	…AAGCCAATAAA…
*Pongo pygmaeus*	…TTGGTCGAAGT…	…TAAACATCTTT…	…AAGCCAATAAA…
*Macaca mulatta*	…TTGGCCGAAGT…	…TAAACATCTTT…	…AAGCCAATAAA…

SNPs rs7206629 at 52378914, rs7202116 at 52379116 and rs7202296 at 52379191 in the human sequence are shown in bold.

We analysed the 420 HapMap Phase II phased SNP haplotypes for CEU, YRI and ASN and found that this region comprises 60 unique haplotypes. A Median-Joining Network analysis [26], [27] (Supplementary Figure S3) was performed, including an inferred ancestral haplotype, which indicated that the susceptibility haplotype is closer to, or less mutational events away from, this ancestral haplotype. This finding suggests that the ability to methylate was gained early, is maintained on the susceptibility haplotype, and has been lost in the younger haplotypes present in humans. However no haplotypic evidence of selection was apparent (XP-EHH [28] or iHS [29], data not shown), although these tests are more powerful for more recent mutational events.

### Enhancer Activity Evidence within the 7.7 kb Region

A ChIP-chip study, using the same T2D array as part of a larger experiment, investigated normal skeletal muscle cells and identified evidence of enhancer activity within the 7.7 kb window, with the classic signature of H3K4me1 and no other strong signal (Supplementary Figure S4). These findings are consistent with those of Ragvin *et al*. who have recently identified long range enhancers within the first intron of *FTO* that influence the expression of *IRX3*, located ∼470 kb downstream of this region. [30]. One of the two critical Highly Conserved Noncoding Elements (HCNEs) proposed to affect *IRX3* expression by enhancer activity that overlaps with risk variants, designated as element 6 by Ragvin *et al*. is located at Chr16:52,377,745–52,378,287, and resides centrally within the broad 7.7 kb peak (Figure 3). This 542 bp element is also only 213 bps 5′ of the 900 bp window.

### Expression Analysis

Direct analysis of allele-specific expression of *FTO* mRNA is not possible as no common coding polymorphisms exist within the LD block, or rest of the gene. Examining the Sanger GENEVAR HapMap CEU expression data identified no significant differences with respect to *FTO* haplotype status in *FTO* or *IRX3* expression from lymphoblastoid cell lines in children or adults [31] (data not shown). We also used an available RNA-Seq dataset derived from the cerebellum of six male individuals [32], but were unable to identify allele-specific expression of *FTO* (by attempting to utilise any pre-mRNA intronic reads that may be present) or *IRX3*. This was due lack of informative SNPs in former and low coverage across the latter.

### T2D versus non-T2D analysis

We also compared individuals with and without T2D, as a case-control analysis, across all the assayed regions of the array, (T2D and obesity related phenotype candidates, 1q linkage region and imprinted regions). We did not identify any disease-related differently methylated regions (DMRs) of statistical significance post false discovery rate correction (data not shown). The average of the variance and standard deviation in methylation level for all 60 individuals across the entire MeDIP data set was 0.02 and 0.14, respectively.

## Discussion

Epigenomic factors, including DNA methylation, histone modifications and non-coding RNAs, are an integral link between DNA sequence variation and subsequent transcriptional output modulation. Increased understanding of these elements will be crucial to obtain a coherent functional assessment of the large number of non-coding DNA variants identified from contemporary whole genome sequencing case-control studies, and to delineate their developmental and tissue-specific features and constraints.

This study investigated the DNA methylation state of genomic loci with evidence for involvement with T2D and found an association with the susceptibility locus that resides within the *FTO* gene. This region was identified in a T2D GWA Study, with a common haplotype located across intron 1, exon 2 and intron 2 captured equivalently by various SNPs, subsequently shown to be mediating its disease susceptibility effect through obesity [22], [33], [34], [35]. Murine models initially lent support to *FTO* itself being the causal gene in the locus; an obesity protective phenotype was observed in the *fto* knock-out mouse [36] and a similar but lesser effect in the hypomorphic *fto* missense I367F mutant [37]. A loss-of-function mutation in *FTO* was recognised in humans as an autosomal-recessive lethal syndrome (OMIM #612938) with a phenotype of multiple malformations and severe growth retardation, with non-obese heterozygote relatives [38]. Attempts to identify expression differences in *FTO* in human skeletal muscle and subcutaneous adipose tissue with respect to risk SNPs have been unsuccessful to date [39] and no evidence of allele-specific expression in immortalised lymphoblastic cell lines has been established [40]. Subsequently Meyre *et al*. identified further *FTO* heterozygous, loss-of-function mutations in obese as well as lean subjects, further clouding *FTO*'s causative role [41] and illustrating the complexity of interpreting the function of this dioxygenase in energy balance [42].

Identification of stable epigenetic modifications may aid the exploration of genotype-phenotype interactions in complex diseases. DNA methylation can exert its functional influence through a range of different processes, including direct effects on transcription factor binding, or indirectly via changes to post-translational histone packaging and modulation of chromatin conformation and function [43]. The ability to detect these epigenetic influences will depend on their direct association with genotypic factors and will therefore range from obligatory to stochastic [44]. Thus we have utilised the power of the large scale GWA studies to look for genotype-methylation state associations.

We have identified a methylation association with the strongly replicated disease haplotype of the *FTO* gene, tagged by SNP rs8050136. Therefore the association identified is with the individuals' genotypes not their particular phenotype status. We confirmed and validated these results at single-base resolution within the contributory signal using bisulphite pyrosequencing. In doing so, we identified that the methylation signal was genetically led by the phase of three CpG-creating SNPs in LD within a narrow 900 bp window peak. We did not find evidence of a *cis*-methylation or hepitype effect [15], [16], [17]. The ∼10% change identified in our MeDIP experiment is likely to be an underestimate in this region as BATMAN calculations are based upon the reference genome. Additional work in murine strains also supports the inference that inherited genetic variability is a major determinate on epigenetic variability [45]. Zhang *et al*. identified ASM with as much as 85% difference between alleles across CpG Islands (CGIs) [46]. However, it is not clear how much of this effect is driven by CpG-creating SNPs or the additional influence on surrounding CpG methylation, other genetic polymorphism effects on the methylation machinery, or a combination of all of these factors. Shoemaker *et al.* have recently observed ASM in 23–37% heterozygous SNPs in differing human cell lines, with 38–88% of these regions dependent on CpG-SNP variation [47]. We have termed our findings of a genetically-driven difference in methylation ability, detected over kilobases, Haplotype-Specific Methylation (HSM), to differentiate this state from epigenetic ASM where methylation will vary between alleles at individual non-SNP CpGs, or a hepitype where genetic variability combines with ASM within a haplotype. In a similarly designed, but larger study, the *FTO* HSM region would be identified as a direct T2D-DMR.

We did not find an association between risk and non-risk haplotypes in the other T2D association LD blocks in this integrated analysis, however this does not exclude the possibility of more subtle effects in these regions. Although the limitation of the MeDIP technique is that is does not enable the evaluation of individual cytosines, it does allow more broad-scale haplotype methylation differences to be identified, such as those driven by CpG-SNPs [14], [47]. These genetic drivers of ASM can be identified in easy accessible tissue, which can then be followed up in the most appropriate disease-related tissue to examine for any surrounding CpG modulation.

Recent work has hypothesized that the lack of evidence for *FTO* expression modulation by susceptibility SNPs may be due to this region having effects on distal surrounding genes including *IRX3* [30] and *RBL2* [48]. Ragvin *et al*. used comparative genomics to identify HCNEs and overlying genomic regulatory blocks, and proposed that enhancers in the first intron susceptibility region exert long range regulatory effects on expression of the developmental transcription factor gene *IRX3*, Iroquois Homeobox 3 located in a gene desert ∼170 kb 3′ of *FTO* [30]. Enhancers are located predominantly in intergenic or intronic regions and may act as regulators of gene transcription over long distances [49], have an activating function on chromatin structure [50], are sensitive to CpG methylation [51] and have a important role in developmental processes [43], [52], [53]. Of two HCNE-containing elements with enhancer effects implicated with a metabolic role, one is located within the 7.7 kb methylation window (chr16:52,371,700–52,379,399). Higher regional methylation of this enhancer, caused through increased methylatable CpG sites, on the susceptibility haplotype may impede its action in terms of enhancer-specific transcription factor recruitment, subsequent chromatin DNA looping, enhancer-promoter interaction and enhanceosome formation [51] with subsequent down-regulation on *IRX3* expression. Additionally this HCNE is just over 200 bp 5′ of the 900 bp window (chr16:52,378,500–52,379,399). Therefore the 900 bp peak is within a 2 kb ‘shore’ region of this enhancer and it may be possible that these ‘Enhancer shores’ act in a similar fashion to ‘CpG Island shores’ (2 kb either side of Islands) and regions of low CpG density, which have been identified with more dynamic DNA methylation effects [54]. Our ChIP-chip data from skeletal muscle indicate a H3K4me1 signature within the 7.7 kb region, as well ChIP-Seq data from cell lines confirms a 5K block of H3K4me1 enrichment completely encapsulated here (http://bioinformatics-renlab.ucsd.edu/enhancer) (Supplementary Figure S4) [50] and a recent examination of histone modifications in pancreatic islets also identified this enhancer marker 1.2 kb wide over rs8050136 within the region [55]. No evidence of allele-specific expression was identified from different sources; therefore whilst the DNA methylation state of the enhancer-including haplotype may be observed in all tissues, due to being predominately genetically driven due to CpG-SNPs, the possible outcome of effect on expression may only be seen in precise cell types at a precise time and/or environmental-specific manner [40].

Despite the interesting genomic overlap between the 7.7 kb region of HSM and the HCNEs identified by Ragvin *et al*., the phylogenetically distant zebrafish knock-down of the orthologous *irx3a* has reduced pancreatic β insulin- and α glucagon-secreting cells and increased ghrelin-producing ε cells. The role of *IRX3* in pancreatic development is in conflict with the knowledge that most obese individuals display an increase in pancreatic beta cell mass as a compensatory response to the peripheral insulin resistance that co-exists [56] and the knowledge that most previously-identified obesity genes are involved in neuronally-mediated central energy balance [42]. However the evidence of functional enhancer capability of this conserved non-coding region is the crucial finding, as its downstream target may have changed or evolved to take on a more complex role over time. It is possible that *IRX3*'s role in neurodevelopment of the posterior forebrain in mammals, including the hypothalamus, may in fact be critical [54], [57], [58]. Redressing the previous evidence in favour of *FTO* causative role, the *fto* knock-out mouse targeted exon 2 and 3, only ∼1 kb into intron 1 [36], therefore did not remove any of the putative enhancers. If *FTO* is involved in the phenotype, the observed methylation change could affect expression by changing gene-body methylation or influencing the isoform balance by modifying exon inclusion or exclusion [59].

Loss or gain of CpG dinucleotides over evolutionary time leading to a genetically-driven variation in DNA methylation and subsequent higher variance has been proposed to be a major driver in evolutionary adaption as well as disease susceptibility [60], [61]. The loss of a CpG site, by deamination of methylated cytosine, can not only can have considerable influence on regional methylation [17], but is also an important mechanism in the formation of transcription factor binding sites [62], such as for p53 that has a role in regulation of insulin resistance [63]. Acquiring the ability to methylate by a cluster of in-phase alleles within a regulatory domain could also be selected for, if functionally significant.

Trans-ethnic studies, especially in genetically diverse African-derived populations, can be informative in narrowing down the location of a causative variant in regions of strong LD in the initial study population [64]. The SNP rs3751812 was the only to confer significant risk (T allele as on CEU susceptibility haplotype, Figure 1) in an African-American study [65] and is in a LD block in two African HapMap populations overlapping the 7.7 kb window.

In conclusion, we have identified variant-CpG restricted Haplotype-Specific Methylation within the *FTO* obesity susceptibility locus. Previous association SNP findings were equivalent across this region and therefore could be consistent with a difference in CpG methylation capability being the driving factor that is inherent to this haplotype. To our knowledge, this is the first identification of any association with a measureable methylation difference within a GWA Study SNP association locus. Detailed analysis of the methylation signal and pyrosequencing validation indicate the genetic phase of CpG-creating SNPs are a strong influence in this finding, indicating LD with CpG-creating SNPs as a relevant consideration in genomic methylation studies. A region of 7.7 kb drove the most significant haplotype-specific methylation, and overlies a region containing putative enhancer sequence. Our observation of increased methylation ability at this enhancer region may contribute towards reducing the efficiency of this regulatory element [51]. Thus the investigation of epigenetic variation may be very useful in narrowing down significant regions in large LD association blocks and proposing functional hypotheses for subsequent follow-up from GWA studies.

## Materials and Methods

### Subjects

Whole blood genomic DNA from Caucasian individuals living in the UK recruited to two pre-existing population cohorts (coordinated by the same research centre) was used for the experimental analyses. Forty UK Warren 2 T2D female participants were selected from trios and had a diagnosis of Type 2 diabetes made by either current prescription of a diabetes-specific medication or laboratory evidence of hyperglycemia (World Health Organization definition) [66]. Forty female participants without diabetes were selected from the Exeter Family Study of Childhood Health and had normal fasting glucose and/or HbA1c levels. The mean age of participants was 37 years (standard deviation 6.6) and mean body mass index 29.4 kg/m^2^ (standard deviation 8.1). Four hypothalamus and ten prefrontal cortex samples were selected from ‘normal brain’ post-mortem tissues from the MRC London Brainbank for Neurodegenerative Diseases at the Institute of Psychiatry in London, and DNA was extracted using standard phenol: chloroform techniques. Written informed consent was obtained from all participants. Ethics approval was covered by the Southwest Multicentre Research Ethics Committee MREC/00/6/55 (UK Warren 2 T2D samples) and Royal Devon and Exeter Healthcare NHS Trust Study 1104 (Exeter Family Study). Post-mortem brain tissue subjects are approached in life for written consent for brain banking, and all tissue donations are consented, collected and stored following legal and ethical guidelines (NHS reference number 08/MRE09/38). The HTA license number for the brainbank is 12293.

### Array Design

The Nimblegen© array comprised of 387,835 50-75mer Probes divided into 122 regions covering 37,037,978 bases of the genome. (080314_HG18_PA_Tiling). This was targeted at all then known T2D Association Loci (20 regions: ^+^/_−_ 60 kb around gene or LD block around association SNP in non-genic regions), Monogenic Obesity & Diabetes (13 genes), the T2D Chromosome 1q linkage region (1q21-24: 148.4–171.3 Mb) comprising 22.8 Mb, identified in multi-continental populations including European, East-Asian, Native-American, and African-American [67]), all Known Imprinted Genes & Imprinting Control Regions from the Imprinted Gene and Parent-Of-Origin Effect Database (www.otago.ac.nz/IGC) gene or control region +/− 5000 bp numbering 82 loci [23] which includes confirmed human, as well as regions syntenic to mouse imprinted loci which have not yet been confirmed in human. Finally, a miscellaneous group of nine loci including Coronary Artery Disease and Stature, hyperuricaemia association regions and the *PPARGCA1* gene [68] were included. For full list see Supplementary Table S1. All coordinates are for genome build NCBI Hs36.1/HG18.

### MeDIP

The protocol was modified from Weber *et al*. [20] as previously described. 1.7 µg DNA is sonicated to produce 300-700 bp fragments. 500 ng DNA is set aside as the ‘input’ control and the remaining 1.2 µg is denatured to become the ‘MeDIP’ fraction. Each denatured DNA sample was incubated with 5MeC-mAb and added to Dynabeads® (Invitrogen). ‘MeDIP’ and ‘input’ DNA are then amplified using the Sigma-Aldrich GenomePlex® Whole Genome Amplification kit. Quality control for MeDIP enrichment efficiency is performed by Q-PCR comparison of two differentially methylated regions of the genome (selected from the Human Epigenome Project primer sequences; methylated locus ID: 6583, unmethylated: 8804; available from www.epigenome.org) with a minimum of a 3 cycle lag for the input compared to the MeDIP fraction for the methylated locus.

### Quality Control

Intensity and Spatial Plots were drawn. Lowess Normalisation was used to smooth out data variation arising from non-linear responses during labelling, hybridisation, or scanning to the two different dye colours. Quantile Normalisation was performed to correct for variation in probe level intensities between arrays. The normalised and raw data are available from GEO (Gene Expression Omnibus, NCBI) under the accession number GSE20553.

### Estimation of Absolute Methylation

Array IP/intensity is assumed to be proportional to the density of methylated CpGs. A Bayesian estimation of methylation within fragments of varied CpG density is made using BATMAN (described in detail in Down *et al.*), with the data output resulting in 100 bp window methylation scores [69]. The algorithm was run individually on each sample.

### Differential Methylation Calling

BATMAN reported methylation values for 100 bp regions, called “proxies”, within larger regions of interest (ROI) varying from 500 to 4000 bp in length. We aimed to call DMRs at the length scale of ROIs to confer with normal methylation consistency that occurs over 500–1000 bp. However, we also observed that some proxies within ROIs exhibited significant variation in methylation. We therefore sought an algorithm that would call DMRs at the length of ROIs, but would use the methylation values of the individual proxies within a ROI rather than an average across all proxies. We adapted an empirical Bayesian method [70], where the case/control status of a sample s (Ps = 1 for case, Ps = 0 for control) is modelled as
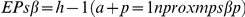
where h is the logit-link function (logistic regression), nprox is the number of proxies in the ROI, and mps is the methylation value for proxy p in sample s. Because the number of samples is not large (∼number of proxies) we simplify the problem by assuming that all regression coefficients are drawn from an underlying distribution with mean zero and finite variance σ2. The null hypothesis
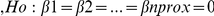
then becomes Ho: σ2 = 0 [70]. The Bayesian algorithm reports *p*-values for rejecting the null-hypothesis for each ROI. To correct for multiple testing, we estimated the False Discovery Rate (FDR) using two approaches: the q-value approach [71] and a permutation-based approach whereby sample labels were permuted a large number of times (>1000).

### LD Block Methylation and Sliding Windows Analysis

Linkage Disequilibrium blocks around genotyped susceptibility genes were defined as per Gabriel *et al*. [18], as implemented in HAPLOVIEW v.4.1 [72]. At each block, subjects were grouped by genotype without reference to case or control status, for each T2D susceptibility SNP. Average methylation score was calculated per block by summating scores for all BATMAN windows within in it and dividing by the number of windows. Non-parametric (Kruskal-Wallis) and parametric (Linear Regression) tests generated *p* value statistics for the mean methylation score with respect to genotype status. Permutation empirical *p*-values were calculated by retaining observed methylation scores and shuffling genotype assignment 10,000 times.

A sliding windows analysis was performed across these LD blocks using 100 bp BATMAN methylation output windows, similar to that used in the context of contiguous SNP haplotypes [73]. Starting with a window size of one and moving one window along per calculation across the entire block, Kruskal–Wallis and Linear Regression analyses were performed for the genotype groups with respect to methylation scores. Window size was increased by one on each pass and the analysis repeated, until the window size equalled the entire LD block. The resulting *p*-values were outputted and plotted at the midway point for each window. Detection of both non-linear and a biologically plausible linear association, between genotype and methylation status provides robust support to the significant relationship at the *FTO* LD block (Supplementary Video S1 and S2). Sliding windows analysis, statistical calculation, and permutation scripts were written with the R package [74].

### Pyrosequencing

Primers were designed using the Biotage PSQ assay design software 1.06 (© Biotage). Primer sequences and PCR reaction details are available from the authors on request. The reaction was performed on the Biotage PSQ HS96 instrument, as per manufactures instructions.

### Bioinformatic Analysis

All genome co-ordinates are given for NCBI Build Hs36.1/UCSC hg18. Scripts for analysis and mining of non-ancestral methylation creating SNPs from dbSNP were written in Perl.

### Median Network Haplotype Analysis

Analysis was performed on phased SNP data from HapMap Phase II SNP (420 phased haplotypes from the CEU, YRI & ASN populations), plus the addition of the inferred ancestral haplotype from ENSEMBL SNP data/dbSNP, for the *FTO* susceptibility LD Block with the NETWORK 4.5.1.0 software [26] (© 2008 Fluxus Technology Ltd). Modelling the hypermutability of CpG transition between 2x to 15x baseline rate did not change tree pattern, only shortened intervening branches with increasing rate (Supplementary Figure S3 is shown for 15x) [75], [76].

### RNA-Seq Analysis

RNA-Seq data generated by an Illumina Genome Analyser from RNA derived from <24 hr post-mortem Cerebellum tissue samples of six anonymous unrelated donor males was available from the paper of Wang *et al*. [32]. These data were aligned using TopHat 1.0, which incorporates the Bowtie aligner and additionally generates splice junction reads [77]. SAM output files were visualised with the Integrative Genomics Viewer (version 1.4.01, http://www.broadinstitute.org/igv).

### ChIP-Chip

Chromatin Immunoprecipitation for H3K4me1, H3K4me2, H3K4me3, CTCF, H3K9me1 and H3K9me2 was performed on the same T2D designed arrays in normal human skeletal muscle cells, as part of a larger experiment utilising a standard protocol [78]. ChIP Peaks were located by MPeak [79]. Duplicates were performed for all, with 90–95% agreement between replicates for all antibodies.

## Supporting Information

Figure S1 FTO HapMap CEU Linkage Disequilibrium. Location of FTO Association LD block indicated by Red Rectangle, as visualised in HAPLOVIEW with LD block as defined by Gabriel et al. [18].
(8.73 MB TIF)

Figure S2 Linear Regression Slope viewed with Linear Regression p-values. Plot of Negative Slope of Linear Regression for the 9 window across the LD block (below) indicating Negative Slope at regions of p-value peaks (above).
(0.43 MB TIF)

Figure S3 Median-Joining Network of FTO susceptibility region haplotypes. Evolutionary relations of the 60 distinct haplotypes from 420 phased haplotypes from HapMap phased haplotypes (CEU, YRI and ASN) plus the Ancestral haplotype. Blue (methylation capable haplotypes within the 900 bp narrow peak with possession of the rs7202116 G allele) and Yellow (non-methylation capable) circles are proportional in size to the number of copies of that haplotype. Lines joining haplotypes are proportional to the number of mutational events separating them. Red nodes are unseen haplotypes, within this sampled set, that are inferred by the MJ algorithm [26]. The thick blue outlined circle represents the haplotype identical to entire CEU susceptibility haplotype indicated in Figure 1 (made up of 49 CEU, 6 YRI & 9 ASN haplotypes).
(8.48 MB TIF)

Figure S4 Enhancer Prediction in 7.7 kb Broad Peak. Enhancer prediction within the Haplotype-Specific Methylation broad peak window of 7.7 kb. Enhancer prediction from H3K4me1 Chip-Seq from Heintzmans et al. in red (http://bioinformatics-renlab.ucsd.edu/enhancer) [50]. Location of Ragvin et al. predicted enhancer (black with red border) and the H3K4me1 data from skeletal muscle indicated in the H3K4me1_SO2 row. Blue rectangle indicates 900 bp differential methylation region window that lies in the shore region of the Ragvin et al. enhancer. CpG creating SNPs in the 900 bp window are indicated with red rectangles. Highest Vertebrate PhastCons Conserved Elements LOD score [81] is underlined in red.
(6.64 MB TIF)

Table S1 Array Design.
(0.05 MB XLS)

Video S1 Sliding windows for Kruskal-Wallis analyses across FTO LD block.
(1.05 MB MOV)

Video S2 Sliding windows for Linear Regression analyses across FTO LD block.
(0.72 MB MOV)

## References

[1] Prokopenko I, McCarthy MI, Lindgren CM (2008). Type 2 diabetes: new genes, new understanding.. Trends Genet.

[2] Dupuis J, Langenberg C, Prokopenko I, Saxena R, Soranzo N (2010). New genetic loci implicated in fasting glucose homeostasis and their impact on type 2 diabetes risk.. Nat Genet.

[3] McCarthy MI, Hirschhorn JN (2008). Genome-wide association studies: potential next steps on a genetic journey.. Hum Mol Genet.

[4] Hu FB, Manson JE, Stampfer MJ, Colditz G, Liu S (2001). Diet, lifestyle, and the risk of type 2 diabetes mellitus in women.. N Engl J Med.

[5] Rakyan VK, Beck S (2006). Epigenetic variation and inheritance in mammals.. Curr Opin Genet Dev.

[6] Beck-Nielsen H, Vaag A, Poulsen P, Gaster M (2003). Metabolic and genetic influence on glucose metabolism in type 2 diabetic subjects—experiences from relatives and twin studies.. Best Pract Res Clin Endocrinol Metab.

[7] Gluckman PD, Hanson MA, Cooper C, Thornburg KL (2008). Effect of in utero and early-life conditions on adult health and disease.. N Engl J Med.

[8] Heijmans BT, Tobi EW, Stein AD, Putter H, Blauw GJ (2008). Persistent epigenetic differences associated with prenatal exposure to famine in humans.. Proc Natl Acad Sci U S A.

[9] Jirtle RL, Skinner MK (2007). Environmental epigenomics and disease susceptibility.. Nat Rev Genet.

[10] Edwards CA, Ferguson-Smith AC (2007). Mechanisms regulating imprinted genes in clusters.. Curr Opin Cell Biol.

[11] Esteller M (2007). Cancer epigenomics: DNA methylomes and histone-modification maps.. Nat Rev Genet.

[12] Javierre BM, Fernandez AF, Richter J, Al-Shahrour F, Martin-Subero JI (2010). Changes in the pattern of DNA methylation associate with twin discordance in systemic lupus erythematosus.. Genome Res.

[13] Wallace C, Smyth DJ, Maisuria-Armer M, Walker NM, Todd JA (2010). The imprinted DLK1-MEG3 gene region on chromosome 14q32.2 alters susceptibility to type 1 diabetes.. Nat Genet.

[14] Schalkwyk LC, Meaburn EL, Smith R, Dempster EL, Jeffries AR (2010). Allelic skewing of DNA methylation is widespread across the genome.. Am J Hum Genet.

[15] Murrell A, Rakyan VK, Beck S (2005). From genome to epigenome.. Hum Mol Genet 14 Spec No.

[16] Yamada Y, Watanabe H, Miura F, Soejima H, Uchiyama M (2004). A comprehensive analysis of allelic methylation status of CpG islands on human chromosome 21q.. Genome Res.

[17] Kerkel K, Spadola A, Yuan E, Kosek J, Jiang L (2008). Genomic surveys by methylation-sensitive SNP analysis identify sequence-dependent allele-specific DNA methylation.. Nat Genet.

[18] Gabriel SB, Schaffner SF, Nguyen H, Moore JM, Roy J (2002). The structure of haplotype blocks in the human genome.. Science.

[19] Christensen BC, Houseman EA, Marsit CJ, Zheng S, Wrensch MR (2009). Aging and environmental exposures alter tissue-specific DNA methylation dependent upon CpG island context.. PLoS Genet.

[20] Teschendorff AE, Menon U, Gentry-Maharaj A, Ramus SJ, Weisenberger DJ (2010). Age-dependent DNA methylation of genes that are suppressed in stem cells is a hallmark of cancer.. Genome Res.

[21] Rakyan VK, Down TA, Maslau S, Andrew T, Yang TP (2010). Human aging-associated DNA hypermethylation occurs preferentially at bivalent chromatin domains.. Genome Res.

[22] Frayling TM, Timpson NJ, Weedon MN, Zeggini E, Freathy RM (2007). A common variant in the FTO gene is associated with body mass index and predisposes to childhood and adult obesity.. Science.

[23] Glaser RL, Ramsay JP, Morison IM (2006). The imprinted gene and parent-of-origin effect database now includes parental origin of de novo mutations.. Nucleic Acids Res.

[24] Arndt PF, Petrov DA, Hwa T (2003). Distinct changes of genomic biases in nucleotide substitution at the time of Mammalian radiation.. Mol Biol Evol.

[25] Cooper GM, Stone EA, Asimenos G, Green ED, Batzoglou S (2005). Distribution and intensity of constraint in mammalian genomic sequence.. Genome Res.

[26] Bandelt HJ, Forster P, Rohl A (1999). Median-joining networks for inferring intraspecific phylogenies.. Mol Biol Evol.

[27] Helgason A, Palsson S, Thorleifsson G, Grant SF, Emilsson V (2007). Refining the impact of TCF7L2 gene variants on type 2 diabetes and adaptive evolution.. Nat Genet.

[28] Sabeti PC, Varilly P, Fry B, Lohmueller J, Hostetter E (2007). Genome-wide detection and characterization of positive selection in human populations.. Nature.

[29] Voight BF, Kudaravalli S, Wen X, Pritchard JK (2006). A map of recent positive selection in the human genome.. PLoS Biol.

[30] Ragvin A, Moro E, Fredman D, Navratilova P, Drivenes O (2010). Long-range gene regulation links genomic type 2 diabetes and obesity risk regions to HHEX, SOX4, and IRX3.. Proc Natl Acad Sci U S A.

[31] Stranger BE, Forrest MS, Dunning M, Ingle CE, Beazley C (2007). Relative impact of nucleotide and copy number variation on gene expression phenotypes.. Science.

[32] Wang ET, Sandberg R, Luo S, Khrebtukova I, Zhang L (2008). Alternative isoform regulation in human tissue transcriptomes.. Nature.

[33] Dina C, Meyre D, Gallina S, Durand E, Korner A (2007). Variation in FTO contributes to childhood obesity and severe adult obesity.. Nat Genet.

[34] Scott LJ, Mohlke KL, Bonnycastle LL, Willer CJ, Li Y (2007). A genome-wide association study of type 2 diabetes in Finns detects multiple susceptibility variants.. Science.

[35] Scuteri A, Sanna S, Chen WM, Uda M, Albai G (2007). Genome-wide association scan shows genetic variants in the FTO gene are associated with obesity-related traits.. PLoS Genet.

[36] Fischer J, Koch L, Emmerling C, Vierkotten J, Peters T (2009). Inactivation of the Fto gene protects from obesity.. Nature.

[37] Church C, Lee S, Bagg EA, McTaggart JS, Deacon R (2009). A mouse model for the metabolic effects of the human fat mass and obesity associated FTO gene.. PLoS Genet.

[38] Boissel S, Reish O, Proulx K, Kawagoe-Takaki H, Sedgwick B (2009). Loss-of-function mutation in the dioxygenase-encoding FTO gene causes severe growth retardation and multiple malformations.. Am J Hum Genet.

[39] Grunnet LG, Nilsson E, Ling C, Hansen T, Pedersen O (2009). Regulation and function of FTO mRNA expression in human skeletal muscle and subcutaneous adipose tissue.. Diabetes.

[40] Verlaan DJ, Ge B, Grundberg E, Hoberman R, Lam KC (2009). Targeted screening of cis-regulatory variation in human haplotypes.. Genome Res.

[41] Meyre D, Proulx K, Kawagoe-Takaki H, Vatin V, Gutierrez-Aguilar R (2010). Prevalence of loss-of-function FTO mutations in lean and obese individuals.. Diabetes.

[42] O'Rahilly S (2009). Human genetics illuminates the paths to metabolic disease.. Nature.

[43] Lister R, Pelizzola M, Dowen RH, Hawkins RD, Hon G (2009). Human DNA methylomes at base resolution show widespread epigenomic differences.. Nature.

[44] Richards EJ (2006). Inherited epigenetic variation—revisiting soft inheritance.. Nat Rev Genet.

[45] Schilling E, El Chartouni C, Rehli M (2009). Allele-specific DNA methylation in mouse strains is mainly determined by cis-acting sequences.. Genome Res.

[46] Zhang Y, Rohde C, Reinhardt R, Voelcker-Rehage C, Jeltsch A (2009). Non-imprinted allele-specific DNA methylation on human autosomes.. Genome Biol.

[47] Shoemaker R, Deng J, Wang W, Zhang K (2010). Allele-specific methylation is prevalent and is contributed by CpG-SNPs in the human genome.. Genome Res.

[48] Jowett JB, Curran JE, Johnson MP, Carless MA, Goring HH (2010). Genetic Variation at the FTO Locus Influences RBL2 Gene Expression.. Diabetes.

[49] Visel A, Rubin EM, Pennacchio LA (2009). Genomic views of distant-acting enhancers.. Nature.

[50] Heintzman ND, Hon GC, Hawkins RD, Kheradpour P, Stark A (2009). Histone modifications at human enhancers reflect global cell-type-specific gene expression.. Nature.

[51] Schmidl C, Klug M, Boeld TJ, Andreesen R, Hoffmann P (2009). Lineage-specific DNA methylation in T cells correlates with histone methylation and enhancer activity.. Genome Res.

[52] Visel A, Blow MJ, Li Z, Zhang T, Akiyama JA (2009). ChIP-seq accurately predicts tissue-specific activity of enhancers.. Nature.

[53] Murgatroyd C, Patchev AV, Wu Y, Micale V, Bockmuhl Y (2009). Dynamic DNA methylation programs persistent adverse effects of early-life stress.. Nat Neurosci.

[54] Caqueret A, Boucher F, Michaud JL (2006). Laminar organization of the early developing anterior hypothalamus.. Dev Biol.

[55] Bhandare R, Schug J, Le Lay J, Fox A, Smirnova O (2010). Genome-wide analysis of histone modifications in human pancreatic islets.. Genome Res.

[56] Lingohr MK, Buettner R, Rhodes CJ (2002). Pancreatic beta-cell growth and survival—a role in obesity-linked type 2 diabetes?. Trends Mol Med.

[57] Braun MM, Etheridge A, Bernard A, Robertson CP, Roelink H (2003). Wnt signaling is required at distinct stages of development for the induction of the posterior forebrain.. Development.

[58] Lee JE, Wu SF, Goering LM, Dorsky RI (2006). Canonical Wnt signaling through Lef1 is required for hypothalamic neurogenesis.. Development.

[59] Suzuki MM, Bird A (2008). DNA methylation landscapes: provocative insights from epigenomics.. Nat Rev Genet.

[60] Feinberg AP, Irizarry RA (2010). Evolution in health and medicine Sackler colloquium: Stochastic epigenetic variation as a driving force of development, evolutionary adaptation, and disease.. Proc Natl Acad Sci U S A.

[61] Akey JM (2009). Constructing genomic maps of positive selection in humans: where do we go from here?. Genome Res.

[62] Zemojtel T, Kielbasa SM, Arndt PF, Chung HR, Vingron M (2009). Methylation and deamination of CpGs generate p53-binding sites on a genomic scale.. Trends Genet.

[63] Minamino T, Orimo M, Shimizu I, Kunieda T, Yokoyama M (2009). A crucial role for adipose tissue p53 in the regulation of insulin resistance.. Nat Med.

[64] Tishkoff SA, Reed FA, Friedlaender FR, Ehret C, Ranciaro A (2009). The genetic structure and history of Africans and African Americans.. Science.

[65] Grant SF, Li M, Bradfield JP, Kim CE, Annaiah K (2008). Association analysis of the FTO gene with obesity in children of Caucasian and African ancestry reveals a common tagging SNP.. PLoS One.

[66] Zeggini E, Weedon MN, Lindgren CM, Frayling TM, Elliott KS (2007). Replication of genome-wide association signals in UK samples reveals risk loci for type 2 diabetes.. Science.

[67] Zeggini E, Damcott CM, Hanson RL, Karim MA, Rayner NW (2006). Variation within the gene encoding the upstream stimulatory factor 1 does not influence susceptibility to type 2 diabetes in samples from populations with replicated evidence of linkage to chromosome 1q.. Diabetes.

[68] Ling C, Del Guerra S, Lupi R, Ronn T, Granhall C (2008). Epigenetic regulation of PPARGC1A in human type 2 diabetic islets and effect on insulin secretion.. Diabetologia.

[69] Down TA, Rakyan VK, Turner DJ, Flicek P, Li H (2008). A Bayesian deconvolution strategy for immunoprecipitation-based DNA methylome analysis.. Nat Biotechnol.

[70] Goeman JJ, van de Geer SA, de Kort F, van Houwelingen HC (2004). A global test for groups of genes: testing association with a clinical outcome.. Bioinformatics.

[71] Storey JD, Tibshirani R (2003). Statistical significance for genomewide studies.. Proc Natl Acad Sci U S A.

[72] Barrett JC, Fry B, Maller J, Daly MJ (2005). Haploview: analysis and visualization of LD and haplotype maps.. Bioinformatics.

[73] Zaykin DV, Westfall PH, Young SS, Karnoub MA, Wagner MJ (2002). Testing association of statistically inferred haplotypes with discrete and continuous traits in samples of unrelated individuals.. Hum Hered.

[74] Ihaka R, Gentleman R (1996). R: A Language for Data Analysis and Graphics.. Journal of Computational and Graphical Statistics Vol.

[75] Walser JC, Ponger L, Furano AV (2008). CpG dinucleotides and the mutation rate of non-CpG DNA.. Genome Res.

[76] Lynch M Rate, molecular spectrum, and consequences of human mutation.. Proc Natl Acad Sci U S A.

[77] Trapnell C, Pachter L, Salzberg SL (2009). TopHat: discovering splice junctions with RNA-Seq.. Bioinformatics.

[78] Forsberg EC, Downs KM, Christensen HM, Im H, Nuzzi PA (2000). Developmentally dynamic histone acetylation pattern of a tissue-specific chromatin domain.. Proc Natl Acad Sci U S A.

[79] Zheng M, Barrera LO, Ren B, Wu YN (2007). ChIP-chip: data, model, and analysis.. Biometrics.

