# Supplementary figures and images for: Integrated Genetic and Epigenetic Analysis Identifies Haplotype-Specific Methylation in the FTO Type 2 Diabetes and Obesity Susceptibility Locus
Source: PLoS One. 2010 Nov 18;5(11):e14040. doi: 10.1371/journal.pone.0014040 (PMC2987816; doi:10.1371/journal.pone.0014040)

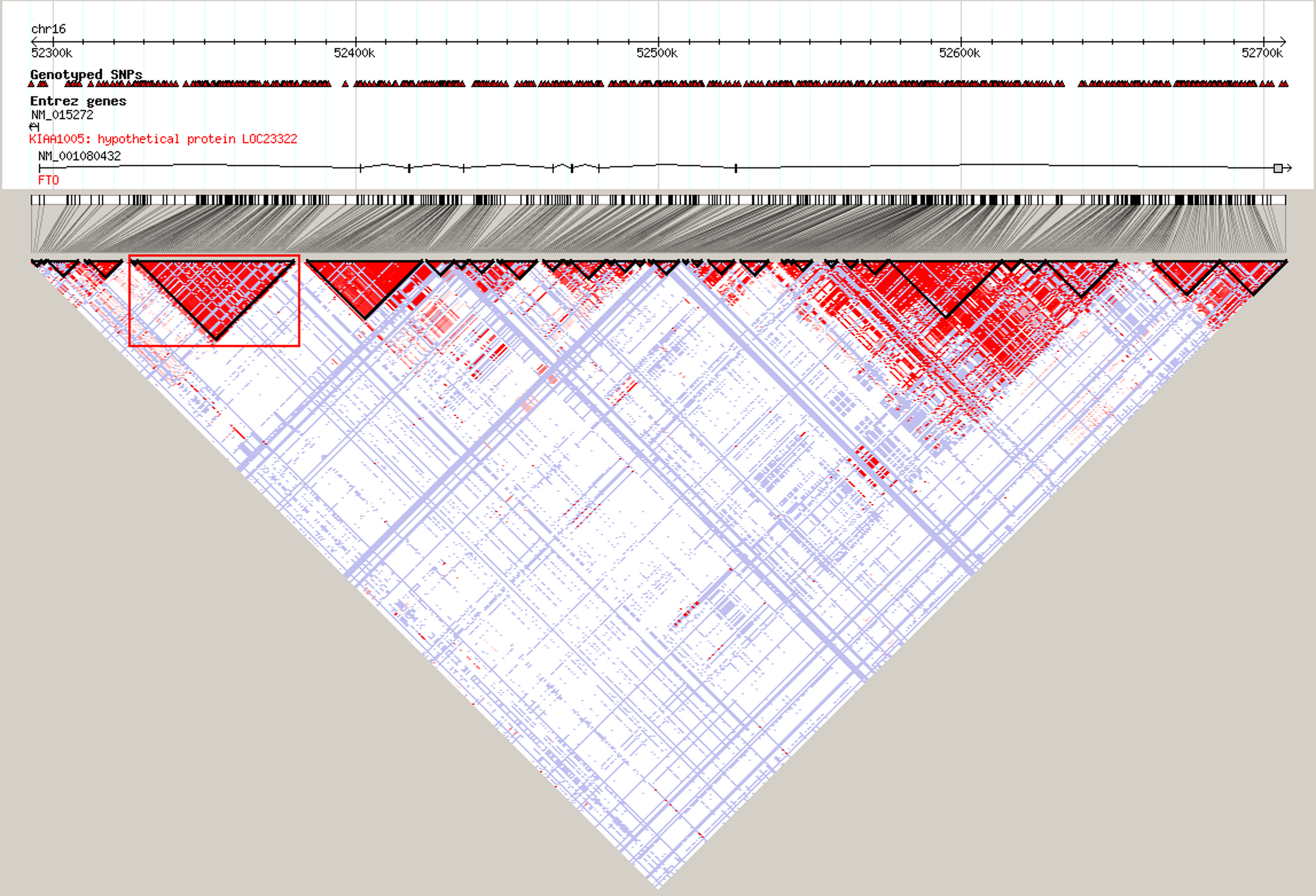

Supplement: Figure S1 — FTO HapMap CEU Linkage Disequilibrium. Location of FTO Association LD block indicated by Red Rectangle, as visualised in HAPLOVIEW with LD block as defined by Gabriel et al. [18]. (8.73 MB TIF) [file pone.0014040.s001.tif]

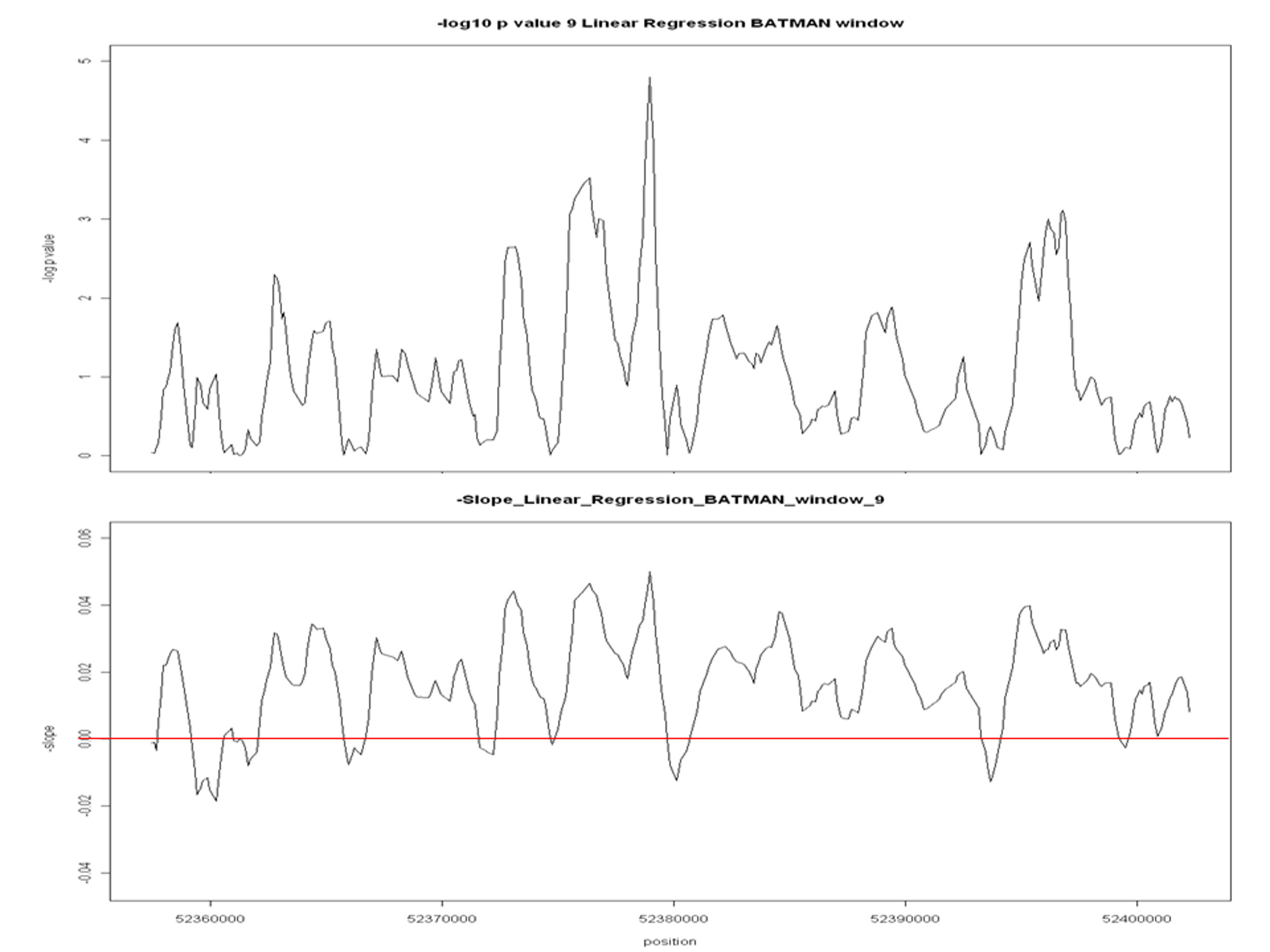

Supplement: Figure S2 — Linear Regression Slope viewed with Linear Regression p-values. Plot of Negative Slope of Linear Regression for the 9 window across the LD block (below) indicating Negative Slope at regions of p-value peaks (above). (0.43 MB TIF) [file pone.0014040.s002.tif]

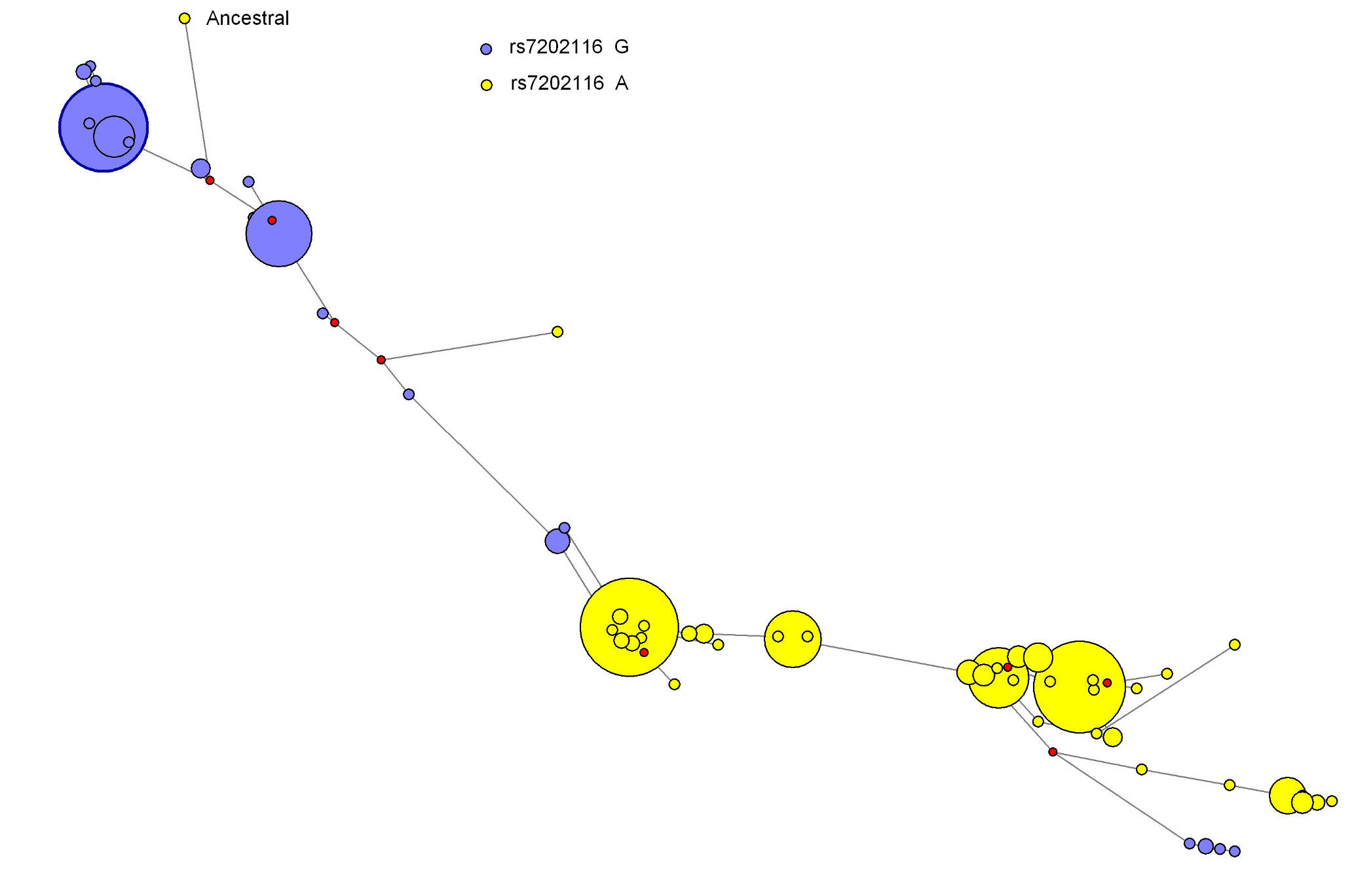

Supplement: Figure S3 — Median-Joining Network of FTO susceptibility region haplotypes. Evolutionary relations of the 60 distinct haplotypes from 420 phased haplotypes from HapMap phased haplotypes (CEU, YRI and ASN) plus the Ancestral haplotype. Blue (methylation capable haplotypes within the 900 bp narrow peak with possession of the rs7202116 G allele) and Yellow (non-methylation capable) circles are proportional in size to the number of copies of that haplotype. Lines joining haplotypes are proportional to the number of mutational events separating them. Red nodes are unseen haplotypes, within this sampled set, that are inferred by the MJ algorithm [26]. The thick blue outlined circle represents the haplotype identical to entire CEU susceptibility haplotype indicated in Figure 1 (made up of 49 CEU, 6 YRI & 9 ASN haplotypes). (8.48 MB TIF) [file pone.0014040.s003.tif]

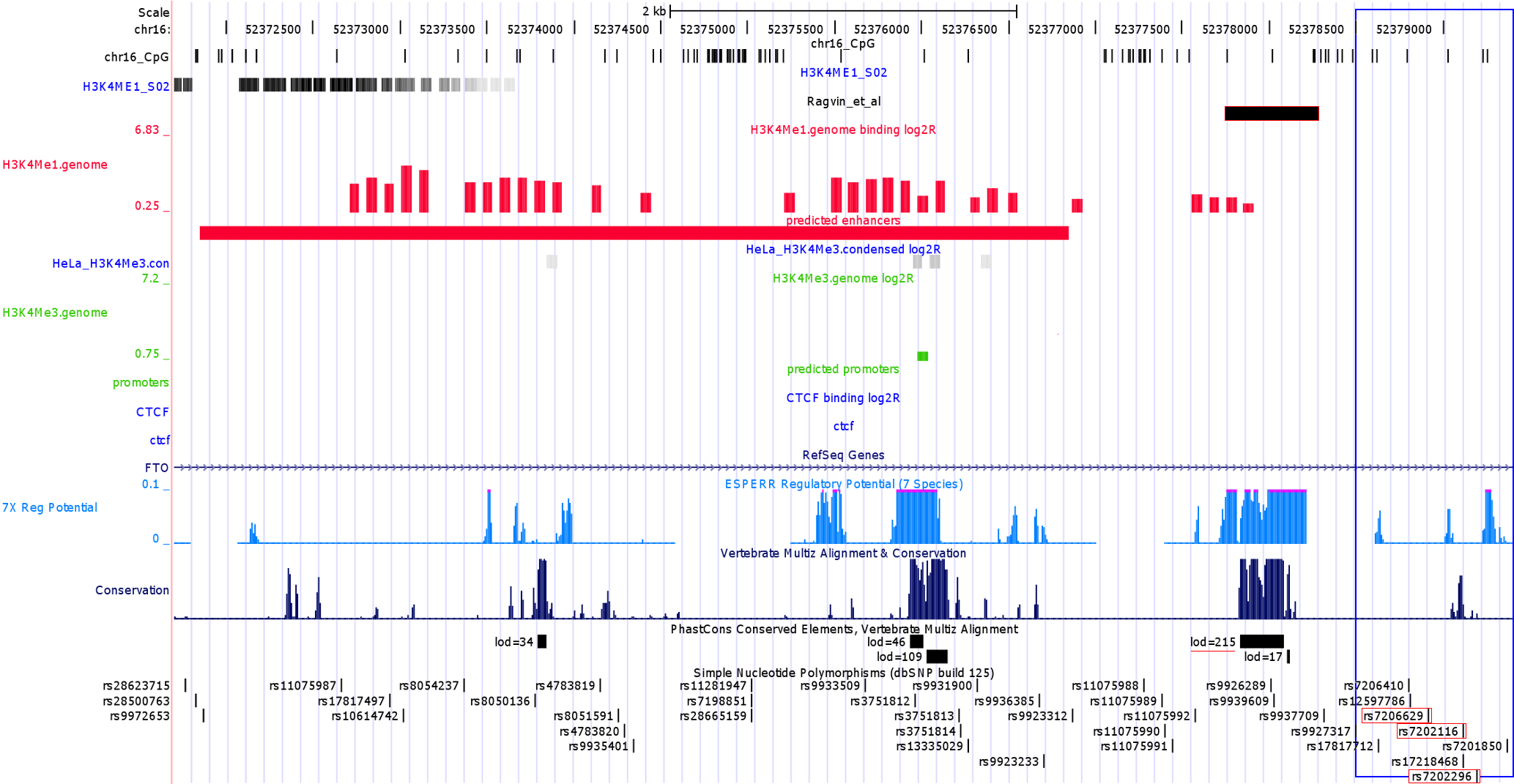

Supplement: Figure S4 — Enhancer Prediction in 7.7 kb Broad Peak. Enhancer prediction within the Haplotype-Specific Methylation broad peak window of 7.7 kb. Enhancer prediction from H3K4me1 Chip-Seq from Heintzmans et al. in red (http://bioinformatics-renlab.ucsd.edu/enhancer) [50]. Location of Ragvin et al. predicted enhancer (black with red border) and the H3K4me1 data from skeletal muscle indicated in the H3K4me1_SO2 row. Blue rectangle indicates 900 bp differential methylation region window that lies in the shore region of the Ragvin et al. enhancer. CpG creating SNPs in the 900 bp window are indicated with red rectangles. Highest Vertebrate PhastCons Conserved Elements LOD score [81] is underlined in red. (6.64 MB TIF) [file pone.0014040.s004.tif]
